# Research Progress on the Interaction Mechanism Between *Morchella* and Mycoparasitic Fungi Causing Diseases and Their Biological Control: A Review

**DOI:** 10.3390/jof12020146

**Published:** 2026-02-17

**Authors:** Ruihua Zhao, Jiayi Xie, Pengfei Jin, Xiaolong He

**Affiliations:** 1 College of Life Sciences, Yan’an University, Yan’an 716000, China; jpf@yau.edu.cn (P.J.); ydhelong@163.com (X.H.); 2 Research and Development Centre of Ecological and Sustainable Application of Microbial Industry of the Loess Plateau in Shaanxi Province, Yan’an 716000, China; 3 School of Medical Technology, Shaanxi Energy Institute, Xianyang 712000, China; xjiayi0428@163.com

**Keywords:** *Morchella*, mycoparasitic fungi, pathogenic factors, response mechanisms, biological control

## Abstract

*Morchella* is a highly valued edible and medicinal fungus with significant nutritional and economic value. In recent years, with the development of artificial cultivation techniques, the planting area of *Morchella* has been expanding continuously, while the incidence of fungal diseases has also increased sharply, seriously affecting its yield and quality and further restricting the development of the *Morchella* industry. To date, ten fungal diseases of *Morchella* have been reported, mainly including white mold, cobweb disease, pileus rot and fungal rot. The mycoparasitic fungi responsible for these diseases can infect *Morchella* by secreting proteins and secondary metabolites, while *Morchella* responds to fungal disease stress through genetic and metabolic regulation. Currently, biological control strategies for *Morchella* fungal diseases primarily rely on antagonistic microorganisms and natural products. This review summarizes the research progress on major fungal diseases of *Morchella*, including their causal fungi, pathogenic factors and infection mechanisms, host response mechanisms, and biological control. It also identifies existing research gaps and prospects for future research directions.

## 1. Introduction

*Morchella* belongs to the phylum *Ascomycota*, class *Pezizomycetes*, order *Pezizales*, family *Morchellaceae*, and genus *Morchella* [1]. Its fruiting body morphology is similar to that of the genus *Peziza* and is characterized by an irregularly shaped pileus and a wrinkled reticulate surface, which resembles a honeycomb structure and a sheep’s stomach in shape [2]. Accumulating studies have confirmed that *Morchella* is rich in proteins, vitamins, and trace elements, with a comprehensive profile of amino acids and a high proportion of unsaturated fatty acids [3]. Moreover, it contains a variety of bioactive components that exert antibacterial, antiviral, tumor-inhibitory, immune-regulating, and anti-fatigue effects, thus establishing it as a highly valued edible and medicinal fungus globally [4,5,6]. Driven by the breeding of new varieties and innovations in cultivation techniques, significant breakthroughs have been achieved in the industrialized cultivation of *Morchella*, further fueling the continuous expansion of its cultivation area and increase in annual output to meet the growing market demand [7,8]. Since 2012, China has successfully realized artificial field cultivation of *Morchella*, and by 2023, its planting area had exceeded 29,000 hectares (ha).

However, with the continuous expansion of *Morchella* cultivation area and the prolongation of its continuous cropping years, the incidence, severity, and taxonomic diversity of fungal diseases have increased year by year. Among these diseases, white mold, cobweb disease, and pileus rot have posed severe threats to the quality and yield of *Morchella* [9,10,11,12]. In particular, *Morchella* is mainly cultivated in seasonal greenhouses, where fungal pathogens spread rapidly and trigger widespread infections in such environments. Approximately 25% of the planting area in China is affected by fungal diseases annually, which has become a major bottleneck restricting the sustainable development of the *Morchella* industry [13,14,15,16]. At present, research findings on *Morchella* fungal diseases are being continuously published, laying a solid foundation for disease prevention and control. Therefore, this paper systematically reviews the major fungal diseases of *Morchella* and their causal fungi, the pathogenic factors and infection mechanisms of the pathogens, the defense response mechanisms of *Morchella*, and the latest progress in biological control technologies. It also identifies existing research gaps and outlines prospects for future research directions, aiming to help researchers quickly grasp the current research status of *Morchella* fungal diseases and provide a theoretical basis for in-depth studies and practical disease management in the future.

## 2. Main Fungal Diseases of *Morchella* and Their Causal Fungi

To date, ten fungal diseases of *Morchella* have been reported worldwide, including white mold, pileus rot, fungal wilt, cobweb disease, fungal rot, stipe rot, white rot, primordia rot, stipe spot, and apothecium deformity. All of these diseases have been confirmed to be induced by fungal pathogens, and their typical symptoms are systematically summarized in Table 1 to facilitate differentiation and accurate identification in practical production. Among these diseases, white mold is the most prevalent and widely distributed, followed by cobweb disease, pileus rot, and fungal rot, which collectively pose the primary threat to *Morchella* cultivation. Fungal diseases of *Morchella* exhibit certain common phenotypic characteristics: obvious white flocculent lesions usually emerge in the early stage, followed by withering of the infected tissues, and ultimately rot and deformation of the fruiting bodies. Notably, under high-temperature and high-humidity cultivation conditions, these fungal diseases are prone to large-scale outbreaks and epidemics, resulting in substantial yield losses [15]. It is worth emphasizing that there are significant symptom similarities among some diseases, posing considerable challenges to field diagnosis and prevention. For instance, the symptoms of pileus rot disease are highly consistent with the white mold symptoms reported by He et al. [13], especially in terms of occurrence conditions, the white mold layer formed on the pileus in the early stage, and the lesion characteristics in the late stage (e.g., dryness, shrinkage, and even perforation). Similarly, white rot and fungal rot also exhibit certain symptom similarities to the field-occurring white mold symptoms, further increasing the difficulty of disease prevention and control [17,18].

Rapid and accurate identification of mycoparasitic fungi is a prerequisite for effective prevention and control of *Morchella* fungal diseases. In recent years, the combined application of morphological observation and PCR-based molecular detection techniques has become the mainstream method for identifying mycoparasitic fungi of *Morchella*. In particular, the analysis of multi-locus gene sequences such as the internal transcribed spacer (*ITS*), translation elongation factor 1-α (*EF1-α*), and the second largest subunit of RNA polymerase II (*RPB2*) has significantly improved the accuracy and reliability of pathogen identification [19]. Based on existing studies, a variety of fungal species have been confirmed as potential pathogens of *Morchella* fungal diseases, including *Paecilomyces penicillatus* [13], *Pseudodiploöspora longispora* [20,21], *Pestalotiopsis trachicarpicola* [22], *Cladobotryum protrusum* [23], *C. varium* [24], *C. mycophilum* [16], *Trichoderma atroviride* [25], *Lecanicillium aphanocladii* [18], *Clonostachys rosea* [26], *Aspergillus niger* [27], *Fusarium incarnatum* [28], *F. equiseti* [28], *F. nematophilum* [29], *F. oxysporum* [30], *Purpureocillium lilacinum* [31], *Cladosporium scabrellum* [32] and *Alternaria alternata* [33] (detailed information is summarized in Table 1). Among these pathogens, Shi et al. [15] collected disease samples from 32 major *Morchella* planting areas in 18 provinces across China, and *ITS* sequence analysis showed that *P. longispora* had a high abundance in almost all samples, indicating its primary role in triggering *Morchella* fungal disease outbreaks. Traceability analysis further revealed that *P. longispora* is widely present in soil and air, and it can be enriched under the specific nutritional or environmental conditions of the *Morchella* cultivation system, then gradually colonize and invade the ascocarps during the fruiting period [15]. In general, the pathogens causing *Morchella* fungal diseases are characterized by high diversity, and mixed infections of multiple pathogens often occur in the field. However, there is no sufficient evidence to indicate significant geographical variation in these pathogens, suggesting that the occurrence of *Morchella* fungal diseases may be more closely related to local cultivation conditions rather than geographical factors.

**Table 1:** Main fungal diseases and pathogens of *Morchella*.

Disease	Typical Symptoms	Pathogen	*Morchella*	Gene Sequence Analysis	Occurring Region and Reference
White mold	White floccose lesions on pileus/stipe; withering, desiccation shrinkage, perforation of fruiting body	*P. penicillatus*	*M. sextelata*, *M. importuna*, *Morchella* sp.	*ITS*	Sichuan [13,34,35,36]; Yunnan [24]; Guizhou [37]
		*P. longispora*	*Morchella* sp., *Morchella* sp. ‘G70’, *M. sextelata*, *M. esculenta*	*ITS*; *ITS*, *BenA*, *CaM*, *LSU*, *SSU*, *TEF* and *RPB2*	Jiangsu [38]; Gansu [39]; Shanxi [21]; Guizhou [40]
Pileus rot	Early-stage white floccose lesions on pileus; late-stage dry shrinkage, crinkling, perforation	*P. longispora*	*M. importuna*, *Morchella* sp.	*ITS*; *ITS* and *LSU*	Guizhou [41]; Hubei [20]
Fungal wilt	White fluffy mycelia on pileus; wilting, rotting, malformation of fruiting bodies	*P. longispora*	*M. importuna*, *Morchella* sp.	*ITS*; *ITS* and *LSU*	Shaanxi [42]; Henan [43]; Chongqing, Hubei [44]
		*P. trachicarpicola*	*M. sextelata*	*ITS*, *TUB* and *TEF-1α*	Sichuan [22,45]
Cobweb disease	Cobweb-like mycelium on fruiting body; rapid spread, withering and death	*C. protrusum*	*M. importuna*	*ITS* and *TEF1*	Shandong [23]
		*C. varium*	*Morchella* sp.	*ITS*	Yunnan [24]
		*C. mycophilum*	*M. sextelata*	*ITS*, *TEF1* and *RPB2*	Guizhou [16]
Fungal rot	Compound fungal infection; soft rot of fruiting body with foul odor	*L. aphanocladii*	*M. sextelata*, *M. esculenta*	*ITS* and *RPB2*	Zhejiang [18,46]
		*T. atroviride*	*M. sextelata*	*ITS*, *TEF1* and *RPB2*	Anhui [25]
		*C. rosea*	*M. sextelata*	*ITS* and *EF-1α*	Anhui [26]
		*A. niger*	*M. sextelata*	*ITS*, *BenA*, *CaM* and *RPB2*	Shanghai [27]
Stipe rot	Stipe base browning; upward spread, softening, lodging, death	*F. incarnatum*–*F. equiseti* species complex	*M. importuna*	*EF-1α*	Henan [28]
		*F. nematophilum*	*M. sextelata*	*ITS*, *RPB2* and *EF-1α*	Sichuan, Henan, Gansu, Guizhou [29]
		*Fusarium* sp.	*Morchella* sp.	*ITS*	Guizhou [47]
		*F. oxysporum*	*Morchella* sp.	*LSU*, *TEF1-α* and *RPB2*	Henan [30]
White rot	Early: similar to white mold; Late: white cottony mycelium, rapid rot	*Aspergillus* sp.	*Morchella* sp.	*ITS*	Henan [17]
Primordia rot	Primordium browning, disintegration; covered with exogenous white mycelium	*P. lilacinum*	*M. rufobrunnea*	*LSU* and *ITS*	Israel [31]
Stipe spot	Brown sunken spots on stipe; severe merging impairs nutrient transport	*C. scabrellum*	*M. importuna*	*ITS*, *EF-1α* and *ACT*	Shaanxi [32]
Apothecium deformity	Apothecium twisting, deformation; abnormal expansion failure	*A. alternata*	*M. importuna*	*ITS*, *LSU* and *RPB2*	Hunan [33]

## 3. Pathogenic Factors and Infection Mechanisms of Two Major Fungal Pathogens Causing *Morchella* Diseases

Investigating the pathogenic factors of fungi infecting *Morchella* is critical not only for deciphering their underlying infection mechanisms, but also for identifying novel candidate targets for antifungal agent development and guiding the design of efficient disease prevention and control strategies. Among the diverse fungal diseases threatening cultivated *Morchella*, white mold and cobweb disease are the most widespread and well-characterized, with their pathogenic pathways being relatively well elucidated; notably, no studies have yet fully characterized the pathogenic factors or infection processes of other reported *Morchella* fungal diseases. Accordingly, this section focuses on these two representative diseases to elaborate on their key pathogenic factors and conserved or distinct infection mechanisms. Genomic analyses have revealed that the mycoparasitic fungi responsible for these two diseases harbor abundant genes encoding secreted proteins and other factors associated with pathogen-host interactions [16]. During host infection, these mycoparasites deploy a repertoire of virulence factors, primarily including secreted proteins and secondary metabolites (SMs), both of which are essential determinants of pathogenicity [48,49]. The key virulence factors identified to date are systematically summarized in Table 2.

### 3.1. White Mold

Secreted proteins are key virulence determinants of mycoparasitic fungi, with core genes in the fungal genome encoding carbohydrate-active enzymes (CAZymes)—enzymes that play vital roles in degrading host cell wall components (e.g., chitin and cellulose) at the early infection stage [50]. Multiple independent studies have reached consistent conclusions regarding the contribution of CAZymes to the pathogenicity of white mold-causing pathogens. Wang et al. [51] identified an extensive repertoire of CAZyme-encoding genes in the whole genome of *P. penicillatus*, with chitinases and β-(1,3)-glucanases representing the dominant classes. Using dual RNA-seq to characterize the interaction between *M. sextelata* and *P. penicillatus*, a subsequent study further verified that numerous CAZyme-encoding genes in *P. penicillatus* were significantly upregulated during infection [52]. Consistently, Chen et al. [35] obtained consistent results through genomic analysis of *P. penicillatus* during its interaction with *M. importuna*. In line with functional characteristics documented in plant pathogenic oomycetes, the expansion of CAZyme gene families in the *P. penicillatus* genome is inferred to improve its parasitic infectivity toward *Morchella* hosts [53,54]. At the initial infection stage, CAZymes and other cell wall-degrading enzymes disrupt the structural integrity of the host cell wall, laying a prerequisite for the successful colonization of mycoparasitic fungi [55]. Further targeted research has clarified that β-glucanases and mannanases are the principal CAZymes in *P. penicillatus* that mediate the degradation process of *M. sextelata* cell walls [52]. Parallel observations in *M. sextelata* infected by *P. longispora* revealed obvious cell wall loosening, followed by cell membrane rupture and leakage of intracellular contents [21]. Collectively, these findings confirm a conserved invasion strategy among white mold-causing pathogens: secreting diverse CAZymes to degrade the host cell wall and establish successful infection [35,52,56].

Beyond CAZymes, additional enzymatic components, including o-phenylenediamine biosynthesis enzymes, aldehyde reductases, and NADPH-hydrate isomerases, have been implicated in regulating the infection process of *P. penicillatus* on *M. importuna* [35]. To characterize the chemical basis of pathogenicity, Dong et al. [56] compared metabolic profiles among *P. longispora*, infected *Morchella*, and uninfected *Morchella* using LC-MS/MS. Four highly abundant peptaibols—longisporin A, septocylindrin B, polysporin B, and alamethicin F-50—were isolated and identified, all of which significantly suppressed mycelial growth of *Morchella* and induced tissue necrosis in treated fruiting bodies. These results firmly establish peptaibols as key virulence factors of *P. longispora* during *Morchella* infection [40]. Additionally, mycoparasitic fungi produce various SMs, including toxins, pigments, and stress-tolerance-related compounds. These SMs facilitate host invasion, nutrient competition, and niche occupation, and are therefore recognized as critical contributors to pathogenicity [56,57]. Genomic analysis of *P. penicillatus* further identified numerous biosynthetic gene clusters associated with cytotoxic secondary metabolites, which are proposed to act synergistically with CAZymes to promote pathogenesis [51]. Despite these advances, several key scientific questions remain unresolved: the synergistic action mechanisms between different CAZymes and peptaibols during infection remain uncharacterized, and the specific molecular targets of peptaibols in *Morchella* cells have not been identified.

### 3.2. Cobweb Disease

Genomic analysis of *C. mycophilum* has uncovered 499 CAZyme-encoding genes, 30 of which belong to the glycoside hydrolase 18 (GH18) subfamily—the most abundant subgroup within the GH superfamily [16]. Given that GH18 encodes chitinase-like proteins, this gene subfamily is hypothesized to mediate the degradation of chitin in *M. sextelata* cell walls during the early infection stage [16,58]. In parallel, the GH55 gene family, which primarily encodes endo-1,3-β-glucanase, has been annotated in the genome of *C. protrusum* and is functionally associated with the degradation of β-1,3-glucan in *M. importuna* cell walls [50]. These convergent findings suggest that cobweb disease-causing pathogens may utilize a conserved early infection mechanism, in which CAZyme-mediated cell wall degradation is a prerequisite for successful host invasion. In addition to CAZymes, most other secreted protein-coding genes in the *C. protrusum* genome are annotated as proteases (peptidases), lipases, hydrophobins, KP4 killer proteins, and ROS-related enzymes [50]. By referencing well-characterized pathogenic mechanisms in other edible mushroom pathogens [59,60], it is reasonable to hypothesize that secreted proteases may selectively degrade defensive enzymes in *Morchella*, while ROS-related enzymes likely attenuate the host’s oxidative stress-induced defense responses during infection [61].

In terms of secondary metabolism, the *aur1* gene in *C. mycophilum* participates in the biosynthesis of yellow fusarubin and a red pigment first characterized in *F. graminearum* [16,62]. Through comparative genomic analysis of *Morchella* pathogens, Xie et al. [40] identified SM gene clusters responsible for the synthesis of leucin A, leucin B, and ochratoxin A in *C. protrusum*, and these SMs are postulated to play pivotal roles in pathogenicity. Notably, mycoparasitic fungi exhibit distinct capabilities in SM biosynthesis: *C. protrusum* possesses a stronger SM synthetic potential than both *P. longispora* and *P. penicillatus*, a divergence that is likely accountable for the interspecific variations in virulence observed among these pathogens [40]. Lan et al. [50] proposed a preliminary mechanistic model for cobweb disease development: pathogens first secrete antifungal compounds (e.g., AAs family enzymes and KP4 killer proteins) to inhibit host cell growth, followed by the secretion of chitinases, endo-1,3-β-glucanases, and other hydrolases (e.g., proteases and lipases) to degrade host cell walls and complete the infection cycle, with hydrophobins playing a key role in regulating this sequential process. However, critical knowledge gaps persist in this field: the precise regulatory pathways governing the biosynthesis and secretion of key SMs remain uncharacterized, the specific molecular functions of hydrophobins during infection have not been experimentally validated, and the synergistic relationships between different pathogenic factors in cobweb disease pathogens remain to be systematically investigated.

**Table 2:** Pathogenic factors in mycoparasitic fungi and their functions.

Disease	Mycoparasitic Fungi	Pathogenic Factor	Function	Reference
White mold	*P. longispora*	Peptaibols	Causing cell wall loosening, cell membrane rupture, tissue necrosis	[56]
	*P. penicillatus*	Chitinase, β-(1,3)-glucanase, antifungal secondary metabolites	Degrading cell wall, inhibiting mycelial growth	[51]
	*P. penicillatus*	Cytotoxic secondary compounds	Synergistic effect in pathogenesis	[51]
	*P. penicillatus*	β-glucanase, mannanase and protease	Participating in host cell wall degradation, penetration, infection	[52]
	*P. penicillatus*	CAZymes	Participating in host cell wall degradation, penetration, infection	[35]
	*P. penicillatus*	Diaminophenylalanine biosynthesis enzyme, aldehyde reductase, NADPH-hydrate isomerase	Participating in host infection regulation	[35]
Cobweb disease	*C. mycophilum*	Glycoside hydrolase GH18 gene family	Degrading chitin in host cell wall	[16]
	*C. protrusum*	Gene family GH55 of endo-1,3-β-glucanase	Degrading β-1,3-glucan in host cell wall	[50]
	*C. protrusum*	Proteases, ROS-related enzymes	Selectively degrading host-secreted defense enzymes and counteracting host stress defense	[50]
	*C. protrusum*	Hydrophobin	Deeply participating in and regulating host infection process	[50]
	*C. protrusum*	Leucin A, Leucin B and Ochratoxin A	Participating in host pathogenicity	[40]

## 4. Response Mechanism of *Morchella* to Pathogen Infection

Under fungal disease stress, *Morchella* regulates the expression levels of various stress-related genes and the activity of transcriptional regulatory factors, which in turn modulate its cellular physiological and biochemical responses. Ultimately, it achieves adaptation to the stress through metabolic adjustments and other mechanisms [63]. In recent years, advances in molecular biology—particularly the application of omics technologies—have paved the way for elucidating the response mechanism of *Morchella* to fungal disease stress, and have also provided an effective approach for breeding disease-resistant *Morchella* varieties to manage these diseases.

### 4.1. White Mold

Numerous studies have investigated the response of *Morchella* to white mold pathogen infection, with the core study parameters (*Morchella* species, mycoparasitic fungi, experimental treatments, technologies, and key response mechanisms) systematically summarized in Table 3. Consistent with omics-based analyses, integrated transcriptomic and proteomic analyses reveal that under *P. longispora* stress, metabolic pathways associated with cell wall and cell membrane metabolism are enriched in *M. sextelata*, with increased activities of chitin synthase I and 1,3-β-glucan synthase—a phenomenon that may contribute to the repair of the host cell wall [21]. In *M. sextelata*, genes encoding chitin recognition proteins and the precursor of the allergen Asp f 15 homolog are upregulated, which activates downstream immune responses. Additionally, genes encoding caffeine-induced death protein 2-domain protein and putative apoptotic proteins are upregulated, whereas cyclin genes are downregulated, and these coordinated expression changes collectively trigger programmed cell death, thereby enhancing the immunity of the fungus [52]. In *M. importuna* infected by *P. penicillatus*, the expression level of cyclin-dependent kinase inhibitor (CDKI) genes is additionally found to increase as the infection progresses. During the early stage of this infection, fatty acid biosynthesis and metabolic pathways are significantly enriched [35]. These pathways have been demonstrated to play a crucial role in host–pathogen fungal interactions [64]. Therefore, glycerolipid metabolism pathways and fungal-pathogen interaction pathways may collectively participate in the response of *M. importuna* to infection by *P. penicillatus* [35]. Notably, there are both commonalities and species-specific differences in the response patterns of different *Morchella* species to white mold pathogens: while programmed cell death and metabolic pathway regulation are conserved responses, the specific genes and pathways enriched (e.g., fatty acid metabolism in *M. importuna* vs. chitin recognition in *M. sextelata*) vary by host species.

In addition, studies have found that genes encoding laccase-2, tyrosinase, and cytochrome P450 were upregulated in *M. sextelata* infected by *P. longispora*, and the tyrosine metabolic pathway was enriched. Among these upregulated genes, the laccase-2 gene exhibits the most pronounced upregulation, which may be involved in the detoxification of toxic metabolites [52,60]. A conserved defensive response across *M. sextelata* to infection by both *P. penicillatus* and *P. longispora* is the induction of melanin-dependent pigmentation, which forms a protective barrier to resist pathogen invasion [21,52]. Excessive lipid oxidation can modify the physical properties of the cell membrane, leading to structural damage. Wang et al. [21] demonstrated that the gene encoding Fet3 (a multicopper oxidase with ferroxidase activity) was upregulated in *M. sextelata* upon *P. longispora* infection, and Fet3 may help prevent cell membrane rupture. Additionally, the significant upregulation of the *Sod2* gene indicates that the antioxidant system plays a crucial role in responding to *P. longispora* infection.

For metabolite-related studies, Su et al. [65] employed LC-MS to investigate metabolite dynamics in *M. esculenta* under *P. longispora* stress. The number of differential metabolites increased significantly as the infection progressed; notably, DL-arginine, proline, L-glutamic acid, D-phenylalanine, trehalose, and ergosterol exhibited substantial alterations. These metabolites are hypothesized to be closely associated with the response of *M. sextelata* to *P. longispora* infection. Liu et al. [37] collected samples of *M. importuna* fruiting bodies at different stages of *P. penicillatus* infection. By analyzing metabolite profile dynamics during the progression of *P. penicillatus*-induced white mold, they observed that the relative abundance of most metabolites decreased with increasing infection severity, a finding that suggests that infection significantly suppresses the production, accumulation, and release of metabolites in *M. importuna* fruiting bodies. Consistent with this finding, Su et al. [65] further verified that disease severity of *P. penicillatus* infection is strongly correlated with the relative abundance of key metabolites in *M. esculenta* fruiting bodies. Specifically, key differential metabolites—including lipids, nucleotides and their derivatives, sugars, organic acids, phenolic acids, and alkaloids—are hypothesized to act synergistically in responding to *P. penicillatus*-induced disease during infection [37].

In summary, *Morchella* primarily responds to white mold infection by regulating cell wall repair, programmed cell death, the antioxidant system, and metabolic reprogramming. For a quick overview and cross-comparison of the experimental design, core parameters, and key conclusions of each study, readers can refer to Table 3. Despite these insights, several knowledge gaps remain: the specific molecular crosstalk between different response pathways (e.g., the antioxidant system and metabolic reprogramming) has not been fully elucidated, and the key regulatory genes coordinating these multi-pathway responses require further identification.

**Table 3:** Mechanism of stress response in *Morchella* to white mold.

Morchella	Mycoparasitic Fungi	Experimental Treatment	Experimental Technology	Response Mechanism	Reference
*M. sextelata*	*P. penicillatus*	Fruiting bodies infected by *P. penicillatus* for 3 and 6 days, respectively, with control group untreated	Illumina Nova-Seq platform, qRT-PCR	Activating downstream immune responses to boost immunity; upregulating laccase-2, tyrosinase & cytochrome P450 genes; enriching tyrosine metabolic pathway; aiding toxic metabolite detoxification; forming melanin protective barrier	[52]
*M. importuna*	*P. penicillatus*	Fruiting bodies infected by *P. penicillatus* for 3 and 6 days, respectively, with control group untreated	Solexa (Illumina HiSeq 2500), SMRT (PacBio RS II), RT-qPCR	Showing increased cyclin-dependent kinase inhibitor gene expression with infection progression; enriching fatty acid biosynthesis and metabolic pathways in early infection	[35]
*M. sextelata*	*P. longispora*	Medium supplemented with *P. longispora* fermentation broth filtrate, control group without the filtrate	Illumina Novaseq 6000 platform, qPCR, Orbitrap Astral LC-MS	Enriching cell wall & membrane-related metabolic pathways; upregulating chitin synthase I & 1,3-β-glucan transferase activities (for cell wall repair); forming melanin protective barrier; enabling the antioxidant system to play a crucial role	[21]
*M. esculenta*	*P. longispora*	Fruiting bodies infected by *P. longispora* for 3 and 5 days, respectively, with control group untreated	LC-MS, PCA, PLS-DA	Showing increased differential metabolite count with infection progression; showing significant differences in DL-arginine, trehalose & sorbic acid (likely closely related to white mold occurrence)	[65]
*M. sextelata*	*P. penicillatus*	Healthy fruiting bodies and three stages of infection	UPLC-MS/MS, PCA, OPLS-DA	Showing decreasing relative content of most metabolites with infection progression; showing potential synergistic response of key differential metabolites (lipids, nucleotides & derivatives, sugars, organic acids, phenolic acids, alkaloids) to the disease	[37]

### 4.2. Other Fungal Diseases

In studies on response mechanisms to other fungal diseases, Li et al. [45] found that infection by *P. trachicarpicola* can induce changes in the activities of host defense enzymes such as superoxide dismutase (SOD), polyphenol oxidase (POD), and catalase (CAT) in *Morchella* fruiting bodies. Infection of *M. rufobrunnea* by *P. lilacinum* induces browning—a phenotypic change that further reflects alterations in oxidase activity in this *Morchella* species under pathogenic infection stress [31]. In contrast, in artificial inoculation experiments, *P. penicillatus*-infected *M. importuna* showed no obvious ascus membrane browning [13]. From the above results (e.g., varying browning degrees under different infections), it can be inferred that *Morchella* exhibits differences in its physiological and biochemical responses when challenged by different pathogen-induced disease stresses. Studies on the response mechanisms of *Morchella* species to fungal disease stress lay a theoretical basis for further identifying disease resistance-related genes, key metabolites, and underlying disease resistance mechanisms in these fungi. Despite this, current research on molecular regulatory mechanisms of responses remains limited to white mold, with fewer studies investigating other fungal diseases infecting *Morchella*. This imbalance highlights a critical research gap: the response mechanisms to non-white mold diseases (e.g., cobweb disease, pileus rot disease) are largely uncharacterized, a gap that hinders the development of comprehensive disease prevention and control strategies for the *Morchella* cultivation industry.

## 5. Biological Control of Fungal Diseases in *Morchella*

Compared with other edible fungi, research on the prevention and control of fungal diseases in *Morchella* started relatively late. Consequently, there are currently no practical and immediately effective measures for fungal disease prevention and control in *Morchella* cultivation [66,67]. Driven by the growing demand for the green and high-quality agricultural development, eco-friendly biological control strategies have emerged as a global research hotspot in the field of fungal disease control—particularly relevant for edible fungi like *Morchella*, as chemical control poses a risk of compromising product quality. To date, a range of biological control strategies have been explored and verified to effectively inhibit the pathogens responsible for *Morchella* fungal diseases, including fungal rot, white mold, and pileus rot. These strategies primarily fall into two categories: (1) beneficial antagonistic microorganisms, including genera such as *Bacillus*, *Pseudomonas*, and *Streptomyces*; and (2) natural products, such as microbial volatile compounds (MVCs) and plant-derived fungistatic agents [36,68,69,70] (Table 4).

### 5.1. Antagonistic Microorganisms

Notably, specific *Bacillus* strains—including *B. subtilis* QST 713, *B. subtilis* ME-1, and *B. amyloliquefaciens* MBI 600—as well as microbial compound inoculants dominated by *Bacillus* species, have all demonstrated significant control efficacy against fungal diseases in edible fungi [71,72,73]. Artificial endosymbiotic associations between *Pedobacter* sp. DDGJ and three *Morchella* strains—*M. sextelata* 13, *M. eximia* SM, and *M. importuna* Y2—can significantly enhance the antagonistic capacity of *Morchella* against the pathogenic fungus *F. oxysporum* and remarkably increase field yield [30]. In contrast to transient biocontrol agents, endophytic bacteria are more suitable for biological control, primarily because they can stably and persistently colonize the internal tissues of the host (without inducing adverse effects)—a trait that allows them to continuously suppress mycoparasitic fungi [74]. As reported by Chen et al. [46], *B. subtilis* strain A9 was isolated from endophytic bacterial communities of *M. esculenta*. In in vitro assays, this strain inhibited *L. aphanocladii* with an antagonistic rate of 72.2%; in field trials, it achieved a disease control efficacy of 62.5% against *L. aphanocladii*-induced diseases in *M. esculenta*. Furthermore, the strain displayed broad-spectrum antagonistic activity against multiple other mycoparasitic fungi infecting *M. esculenta*. Genomic analysis of *B. subtilis* A9 revealed an antibiotic biosynthetic gene cluster [46], which may act by disrupting the fungal cell wall, leading to hyphal damage [75]. Electron microscopy also showed abnormal hyphal development, impaired hyphal integrity, and reduced spore formation in *L. aphanocladii* [46]. Beyond direct antagonism, biocontrol *Bacillus* strains can further activate the expression of disease resistance-related genes in the host, thereby enhancing the host’s intrinsic disease resistance [76,77]. Consistently, transcriptomic analysis by Chen et al. [46] found that *B. subtilis* A9 upregulated the expression of defense enzyme genes such as POD, SOD, phenylalanine ammonia-lyase (PAL), and CAT in *M. esculenta*, regulated nitrogen metabolism, and induced the pentose phosphate pathway (PPP). Since antioxidant enzymes are critical for scavenging reactive oxygen species (ROS) and protecting against oxidative damage [78], these results suggest that *B. subtilis* A9 induces host-induced systemic resistance (ISR) by regulating stress response and redox metabolism genes, indirectly enhancing host resistance to pathogen infection [46]. Collectively, studies on the tripartite interaction among *Morchella*, biocontrol *Bacillus*, and mycoparasitic fungi provide novel insights into the multi-dimensional mechanisms underlying the action of biocontrol antagonistic microorganisms.

*Pseudomonas*, a genus of biocontrol bacteria, has been successfully applied to alleviate soil-borne diseases and is also recognized as a group of strains that can promote the growth of edible fungi [71,79]. A key advantage of *Pseudomonas* is its dominant abundance in the rhizosphere soil microbiota of both wild and cultivated *Morchella*, where it influences key physiological processes of *Morchella*, including mycelial growth, fruiting body development, and nutrient uptake [14,80]. Lohberger et al. [81] have reported that co-culture of *M. crassipes* and *P. putida* improves proteolytic enzyme activity and enhances the hydrolysis capacity for organic nitrogen sources. Thus, modulating the population of *Pseudomonas* may help establish a healthy rhizosphere soil microbiota for *Morchella*, suppress populations of *P. penicillatus*, and alleviate white mold severity [70]. *P. chlororaphis* is a dominant bacterial species in the rhizosphere soil microbiota of *M. importuna* [82]. After inoculating *P. chlororaphis* into the continuous cropping soil of *M. importuna* with a high incidence of white mold, biofilms of *P. chlororaphis* covered the mycelia and ascus surfaces of *M. importuna* [70]—this coverage forms a physical barrier against pathogen invasion [83,84]. Soil metagenomics further revealed elevated abundances of genes encoding alkaline proteases and chitinases (which degrade *P. penicillatus* cell walls) and reduced abundances of genes encoding glucanases and laccases (which aid *P. penicillatus* infection) [70]. Importantly, *P. penicillatus* is ubiquitous in soil but only induces disease under conditions of imbalanced microbial communities or weak host resistance [14]. This highlights a critical research focus: understanding the environmental factors and ecological mechanisms that trigger common soil microorganisms to become opportunistic pathogens is essential for developing targeted control strategies [70]. These studies confirm that regulating the mycosphere microbiota of macrofungi can inhibit microfungal soil-borne diseases, providing a scientific basis for developing *Morchella*-specific biocontrol strains and green control strategies.

In addition, *Streptomyces* species exhibit strong inhibitory activity against edible fungus pathogens [85]. Liu (2024) [86] isolated and screened two antagonistic actinomycetes, *S. rochei* and *S. tricolor*, from the rhizosphere soil of *Morchella*. The fermentation broths of these two strains inhibited the mycelial growth of *P. trachicarpicola*, while their volatile substances suppressed conidia germination—indicating a synergistic antagonistic effect through multiple mechanisms. The fermentation broth also showed high antifungal activity in field trials. However, a critical challenge remains: highly efficient antagonistic strains screened in the laboratory are vulnerable to field environmental fluctuations, making it difficult for them to rapidly colonize the *Morchella* rhizosphere and inhibit pathogens. This poor colonization greatly reduces disease control efficacy, so further research is needed to evaluate their applicability and effectiveness in *Morchella* cultivation systems [67].

### 5.2. MVCs

MVCs exert significant regulatory effects on the growth and development of other microorganisms (both prokaryotes and eukaryotes) and play pivotal ecological roles in maintaining the structural and functional stability of soil microbial communities—a key factor for soil ecosystem health [87,88,89]. 1-Octen-3-ol, a volatile compound produced by macrofungi, has broad-spectrum antifungal activity and exerts dual inhibitory and stimulatory effects on different fungi [90,91]. Berendsen et al. [92] have reported that 1-octen-3-ol can inhibit *Lecanicillium* sp. and effectively control dry bubble disease of *Agaricus bisporus*. Pretreating soil with 1-octen-3-ol prior to sowing *M. sextelata* significantly altered the soil microbial community structure throughout its growth cycle, reducing *P. penicillatus* populations and thereby decreasing white mold incidence [36]. Further analysis revealed an extremely significant positive correlation between soil *Rhodococcus* abundance and *M. sextelata* yield [36]. Analogous to its role in other mushroom systems, *Pseudomonas* stimulates *A. bisporus* primordia formation by metabolizing 1-octen-3-ol [93,94], and *M. crassipes* forms a mutualistic interaction with *P. putida* (bacteria proliferate using fungal exudates, while fungi obtain supplementary carbon sources) [95]. Based on these interspecific interaction patterns, it is hypothesized that *Rhodococcus* also stimulates *M. sextelata* fruiting body formation by metabolizing soil-accumulated 1-octen-3-ol [36]. Systematic investigations into the regulatory relationships between 1-octen-3-ol, *Morchella* fruiting body development, and specific soil bacterial populations have led to the development of a novel soil microecology-based strategy for addressing *Morchella* fruiting failure, providing an ecologically friendly alternative to traditional cultivation approaches.

### 5.3. Phytogenic Antimicrobial Agents

A variety of phytogenic antimicrobial agents—including plant essential oils, aqueous/organic solvent extracts, and natural plant acids—have shown pronounced antifungal activity against edible fungus diseases, providing an environmentally benign alternative to synthetic fungicides [19,96,97]. For example, 20% eugenol at a 2000-fold dilution significantly inhibits *P. longispora* mycelial growth while showing high safety to *Morchella* mycelia [69]. Plants like Chaotian pepper, garlic, Chinese prickly ash, and ginger also exhibit antifungal activity against *Pestalotiopsis* spp. [98]. The antifungal mechanisms of these agents are presumably associated with their volatile and non-volatile metabolites, which disrupt fungal cell wall integrity [99]. Despite their demonstrated in vitro efficacy, current research on phytogenic agents for *Morchella* disease control is limited to in vitro tests. Their vulnerability to environmental factors and poor stability has hindered their practical application in *Morchella* cultivation [67]. Two key knowledge gaps persist: (1) the control efficacy of these phytogenic antimicrobial agents in *Morchella* field cultivation remains unvalidated; (2) as plant extracts are complex mixtures, potential functional antagonism among their components and the underlying control mechanisms of these extracts require further clarification.

**Table 4:** Biological control of fungal diseases in cultivated *Morchella*.

Biological Control		Mycoparasitic Fungi	Result	Reference
Antagonistic microorganisms	*B. subtilis* A9	*L. aphanocladii*	Causing abnormal hyphal development of pathogens & reducing spore formation; activating expression of host defense enzyme genes; regulating nitrogen metabolism; inducing pentose phosphate pathway	[46]
	*P. chlororaphis*	*P. penicillatus*	Forming a biofilm protective barrier on host mycelium & ascus surface; increasing abundance of alkaline protease/chitinase genes (acting on pathogen cell wall); reducing abundance of glucanase/laccase genes (involved in pathogen infection)	[70]
	*S. rochei*, *S. tricolor*	*P. trachicarpicola*	Affecting pathogen mycelial growth (via fermentation broth); influencing pathogen conidia germination (via volatile substances); mechanisms unexplored	[86]
Microbial volatile compounds	1-Octen-3-ol	*P. penicillatus*	Modifying soil microbial community structure to reduce pathogen numbers; increasing *Rhodococcus* abundance; metabolizing soil 1-octen-3-ol to stimulate *Morchella* fruiting body formation	[36]
Phytogenic antimicrobial agents	Eugenol	*P. longispora*	Inhibiting mycelial growth of mycoparasitic fungi; mechanism unexplored	[69]
	Hot pepper, garlic, Chinese prickly ash, and ginger	*Pestalotiopsis* sp.	Exhibiting antifungal effects against mycoparasitic fungi; mechanism unexplored	[98]

## 6. Conclusions and Prospects

**Figure 1:**
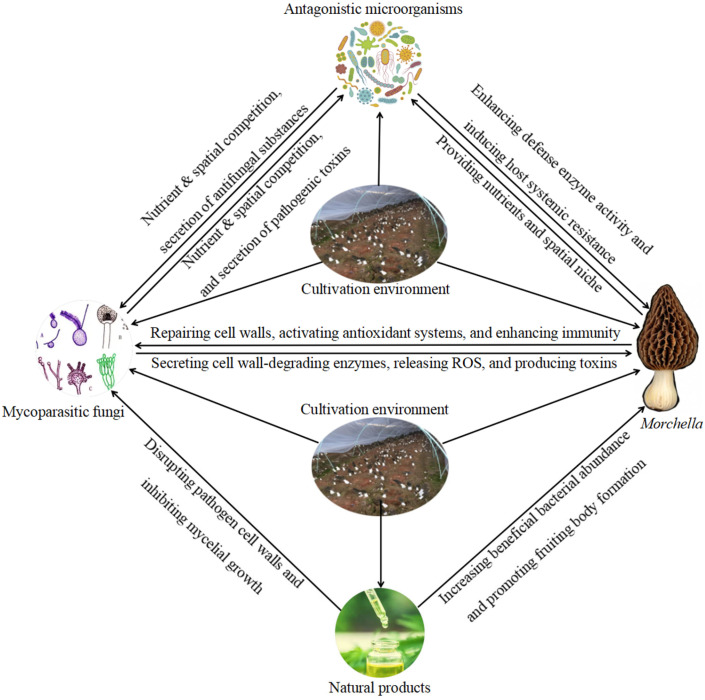
Interactions among antagonistic microorganisms/natural products, mycoparasitic fungi, *Morchella*, and the cultivation environment.

This review provides a comprehensive and detailed overview of the current research status regarding the interaction mechanisms between *Morchella* and fungal pathogens, as well as the biological control of *Morchella* fungal diseases, by synthesizing existing knowledge regarding key aspects, including the major fungal diseases and causal fungi affecting *Morchella*, the pathogenic factors and infection mechanisms of these fungal pathogens, the host defense response mechanisms of *Morchella*, and the currently developed biological control technologies (Figure 1). However, the current research on *Morchella* fungal diseases still has several critical limitations: (1) The lack of large-scale, systematic surveys of *Morchella* fungal diseases has led to insufficient clarity about the primary disease types prevalent in different cultivation regions. (2) Most existing studies on *Morchella* fungal diseases are confined to pathogen identification reports and analyses of soil samples collected from affected cultivation areas, and there is a lack of systematic research on the occurrence dynamics, epidemiological patterns, damage levels, and control strategies of these diseases. (3) Transcriptome sequencing has facilitated the identification of several genes and gene families involved in the immune response of *Morchella*; however, the specific functions of these genes are not fully elucidated. Furthermore, research addressing the pathogenic mechanisms of most causative pathogens and their corresponding host response mechanisms in *Morchella* is still largely unexplored. (4) In commercial production, the prevention and control of *Morchella* fungal diseases remain heavily reliant on agricultural cultivation practices and chemical fungicides, and there are significant gaps in both the application of biological control and related mechanistic research. Consequently, research addressing *Morchella* fungal diseases is relatively underdeveloped—i.e., both in the macroscopic field of disease epidemiology and the microscopic realm of molecular biology—with considerable room for improvement in terms of research breadth and depth.

Moving forward, research on *Morchella* fungal diseases may prioritize the following key directions: (1) For the identification of *Morchella* fungal diseases and their causal pathogens: Conduct comprehensive surveys of diseases across major *Morchella* cultivation regions to define the pathogenic microbial community, thereby clarifying the key diseases prevalent in these areas. For pathogen identification, based on multi-gene sequence analysis, develop rapid and accurate molecular detection techniques—including DNA barcoding, quantitative polymerase chain reaction (qPCR), and loop-mediated isothermal amplification (LAMP). (2) For the interaction mechanisms between *Morchella* and its mycoparasitic fungi: Elucidate the molecular mechanisms underlying pathogen infection and identify the critical stages of disease development, to provide robust support for disease prevention and control. Additionally, accelerate the fine mapping of major disease resistance loci in *Morchella*, the cloning of resistance-related genes, and the dissection of their regulatory mechanisms—all of which will lay a solid theoretical foundation for breeding disease-resistant *Morchella* cultivars and optimizing disease control strategies. (3) In the field of biological control: Sustained efforts should be devoted to screening for potential biocontrol microbial resources. On one hand, endophytes isolated from *Morchella* should be explored for their antagonistic secondary metabolites and growth regulators that promote *Morchella* development, thereby facilitating the development of novel, *Morchella*-specific microbial pesticides. On the other hand, the composition and structure of soil microbial communities in high-yield versus low-yield *Morchella* cultivation soils should be analyzed; based on these insights, composite microbial inoculants can be formulated through rational combination to suppress soil-borne pathogens. Furthermore, transferring antagonism-related genes from highly antagonistic bacterial strains into strains with enhanced environmental adaptability to construct genetically engineered biocontrol agents represents a viable strategy for improving biocontrol efficacy. Additionally, leveraging omics technologies to dissect the multi-dimensional antibacterial mechanisms of biocontrol agents, as well as to decipher the interaction dynamics among the quartet system consisting of the host, pathogen, biocontrol agent, and indigenous microbiota, is critical for developing tailored formulations and application protocols.

## Data Availability

No new data were created or analyzed in this study. Data sharing is not applicable.
